# Predicting variant deleteriousness in non-human species: applying the CADD approach in mouse

**DOI:** 10.1186/s12859-018-2337-5

**Published:** 2018-10-12

**Authors:** Christian Groß, Dick de Ridder, Marcel Reinders

**Affiliations:** 1 Delft Bioinformatics Lab, University of Technology Delft, Van Mourik Broekmanweg 6, Delft, 2600GA The Netherlands; 2 Bioinformatics Group, Wageningen University & Research, Wageningen, 6708 PB The Netherlands

**Keywords:** Genomics, Genome annotation, Variant annotation, Sequence annotation, Mouse genetics

## Abstract

**Background:**

Predicting the deleteriousness of observed genomic variants has taken a step forward with the introduction of the Combined Annotation Dependent Depletion (CADD) approach, which trains a classifier on the wealth of available human genomic information. This raises the question whether it can be done with less data for non-human species. Here, we investigate the prerequisites to construct a CADD-based model for a non-human species.

**Results:**

Performance of the mouse model is competitive with that of the human CADD model and better than established methods like PhastCons conservation scores and SIFT. Like in the human case, performance varies for different genomic regions and is best for coding regions. We also show the benefits of generating a species-specific model over lifting variants to a different species or applying a generic model. With fewer genomic annotations, performance on the test set as well as on the three validation sets is still good.

**Conclusions:**

It is feasible to construct species-specific CADD models even when annotations such as epigenetic markers are not available. The minimal requirement for these models is the availability of a set of genomes of closely related species that can be used to infer an ancestor genome and substitution rates for the data generation.

**Electronic supplementary material:**

The online version of this article (10.1186/s12859-018-2337-5) contains supplementary material, which is available to authorized users.

## Background

With the possibility of determining variation in genomes at large scale came an interest in predicting the influence of a mutation on a phenotype, in particular its pathogenicity. Initially, such predictions were restricted to missense mutations, as these cause a change in the corresponding amino acid chains and are thus most likely to have immediate functional effects. SIFT [1], PolyPhen2 [2], SNAP2 [3] and Provean [4] are examples of this kind of predictor. Recently, a number of methods for variant annotation were proposed that assign a single deleteriousness score to mutations throughout the entire genome, based on a large collection of genomic and epigenomic measurements. These methods – a.o. CADD [5], GWAVA [6], FATHMM-MKL [7] – are based on supervised classification. CADD (Combined Annotation Dependent Depletion) takes an interesting approach, in that it trains classifiers to distinguish between observed benign variants and inferred, putatively deleterious variants, instead of exploiting only known regulatory or disease-associated variants. This opens up the possibility to reproduce this approach for other non-human species as well. It shares similarities with fitCons [8] and LINSIGHT [9] by exploiting evolutionary models, which capture signals of natural selection over many generations in the generation of training data.

Although the use of CADD is already well-established in human genetics research and clinical practice [10, 11], for non-human species the situation is quite different. While generic predictors such as SIFT, Provean and SNAP2 can be used, genome-wide variant annotation methods are generally not available. A major reason is that for non-human genomes fewer genomic annotations are available, complicating the construction of more advanced models. This is even the case for model organisms, such as zebrafish (*Danio rerio*), drosophila (*Drosophila melanogaster*) and mouse (*Mus musculus*). Additionally, extensive population studies similar to the 1000 and 100,000 Genomes Projects [12, 13] are lacking for non-human species, hampering the creation of good training data sets. Finally, models for non-human species are much more difficult to evaluate due to a lack of known disease-associated or phenotype-altering variants such as ClinVar offers for human [14].

Here, we explore the development of a functional prioritization method for SNVs located across the entire genome of a non-human species. The species we selected to investigate is mouse. As a model species it is well studied, with relatively rich, publicly available, genomic annotation data sets [15–20]. Even though not all annotations used in the human CADD model are available for mouse, the large overlap of annotations allows performance evaluation and comparison between the original CADD and our mouse CADD. With this proof-of-principle, we aim to gain insight into design choices for porting such a methodology to non-human species.

## Results

We trained a CADD model on mouse data (mCADD) and a CADD model on human data (hCADD). Performances of both are evaluated on test sets of variants located in different genomic regions. In addition, mCADD is evaluated on three validation sets (Fairfield, Mutagenetix, ClinVar-ESP data sets). We also compared mCADD to benchmark metrics such as SIFT and two PhastCons scores based on two phylogenies of different depth. Further, we trained mCADD and hCADD on four different annotation subsets to investigate the performance of a CADD-like classifier for species with fewer known annotations. These models are referred to as hCADD(n) and mCADD(n), with *n* the number of annotations used during training. To investigate the benefits of developing species-specific CADD models, we compared mCADD to 1) CADD v.1.3. C-scores by lifting validation variants from mm10 to hg19, and 2) a CADD model trained on human data which, without further adaptation, is applied on mouse data to evaluate the mouse SNVs (hCADD*).

### mCADD performs similarly on mouse as hCADD does on human

**Fig. 1:**
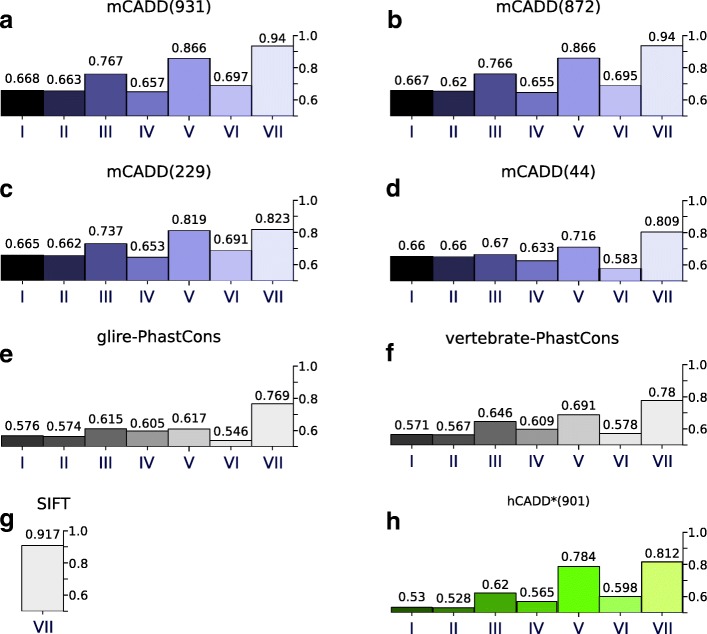
**a**-**d**) ROC-AUC scores of the four different mCADD models evaluated on seven different subsets of the mouse held-out test set reflecting different genomic regions and/or functional annotations. **e**, **f**) Seven different subsets of the mouse held-out test set evaluated by glire- and vertebrate based PhastCons scores, respectively. **g**) Missense mutations of the mouse held-out test set evaluated by SIFT. **h**) The sub sets of the mouse held-out test set evaluated by hCADD*.: I) all data, II), not-transcribed, III) transcribed, IV) transcribed but not translated, V) translated, VI) translated and synonymous, and VII) translated and missense. The different models are indicated at the top of the panel. All displayed scores are ROC-AUC

**Fig. 2:**
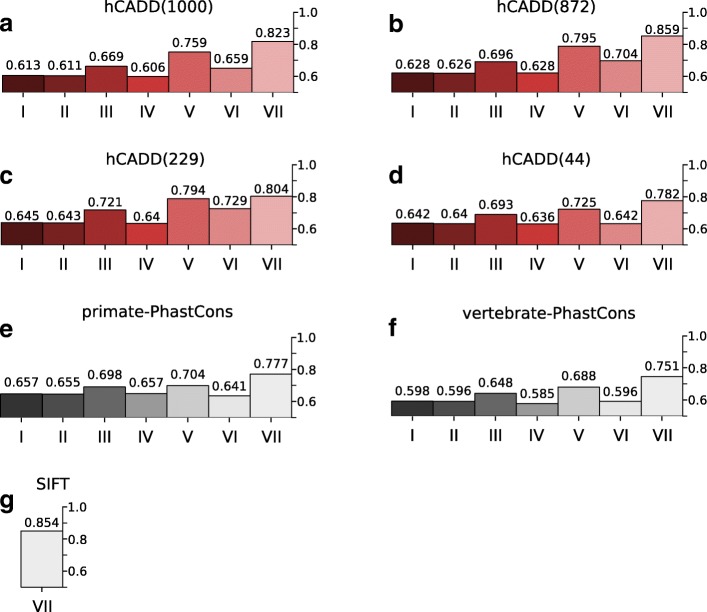
**a**-**d**) ROC-AUC scores of the four different hCADD models evaluated on the human held-out test set. **e**, **f**) Seven different subsets of the human held-out test set evaluated by primate- and vertebrate based PhastCons scores, respectively. **g**) Missense mutations of the human held-out test set evaluated by SIFT. (see caption Fig. 1 for remaining explanation)

The ROC-AUC performance of mCADD(931) on the entire test set equals 0.668 (Fig. 1a), which is similar to the performance of hCADD(1000) applied on human data (Fig. 2a). Overall, mCADD(931) has a better performance across all genomic regions, with the most pronounced difference for the translated missense variants. Both models, mCADD(931) and hCADD(1000), discriminate between simulated and derived better than SIFT and PhastCons scores (see Figs. 1 and 2e-g).

It is known that the distribution of CADD scores differs between genomic regions, and that the disruptive effect of variants in exonic regions can be estimated more precisely than that of variants in non-coding regions [21, 22]. We observe a similar trend for mCADD(931) as well as hCADD(1000). Most of the performance increase from genomic regions I, III, V to VII (Fig. 1a-d) is due to the high performance on correctly classifying missense mutations that become more enriched in these regions. This is in contrast to the performances in genomic regions II, IV and VI which do not contain any missense mutations.

### Models trained on selected annotation subsets experience performance drop in coding regions

To see whether models behave differently when less information is available, we reduced the number of annotations to train human and mouse models. The first subset of annotations (872) was chosen based on the idea that epigenetic measurements and species-specific annotations might not be available for some species. The performances of mCADD(931) and hCADD(1000) as well as mCADD(872) and hCADD(872) are very similar, with the mCADD models performing slightly better than the hCADD models (Figs. 1 and 2).

The second subset of annotations consist of 229 annotations derived from sequence only, i.e. conservation scores and VEP consequences (mCADD(229), hCADD(229)). The situation is now different. The trend is still that performance increases from non-coding to coding to missense mutations. Also, SNVs in non-coding regions can still be classified with a performance comparable to that of models with more annotations. However, with the loss of particular information about coding regions and SIFT as an annotation, the performance of mCADD(229) to evaluate missense mutations drops below that of SIFT.

The smallest subset (44 annotations) excludes the VEP consequences and solely contains conservation scores and sequence features (mCADD(44), hCADD(44)). Now performances drop even further, but mCADD(44) shows that a simple combination of sequence based features and conservation scores outperforms the PhastCons scores for all genomic regions.

Interestingly, hCADD* (the human trained model applied on mouse data) performance lays between mCADD(229) and mCADD(44) for all translated regions (see Fig. 1c, d and h V-VII) and is better than the PhastCons scores for those variant sets. On the other hand, hCADD* shows mostly random performance when non-translated regions are considered, indicating it is necessary to adapt the CADD model to species-specific data.

Taken together, decreasing the number of available annotations decreases performance, which drops relatively faster in coding regions than in non-coding regions. The drop in performance between mCADD(931) and mCADD(872) is, however, negligible, suggesting that epigenetic and species-specific annotations can be safely ignored.

### Evaluation of phenotype affecting SNVs by mCADD

**Fig. 3:**
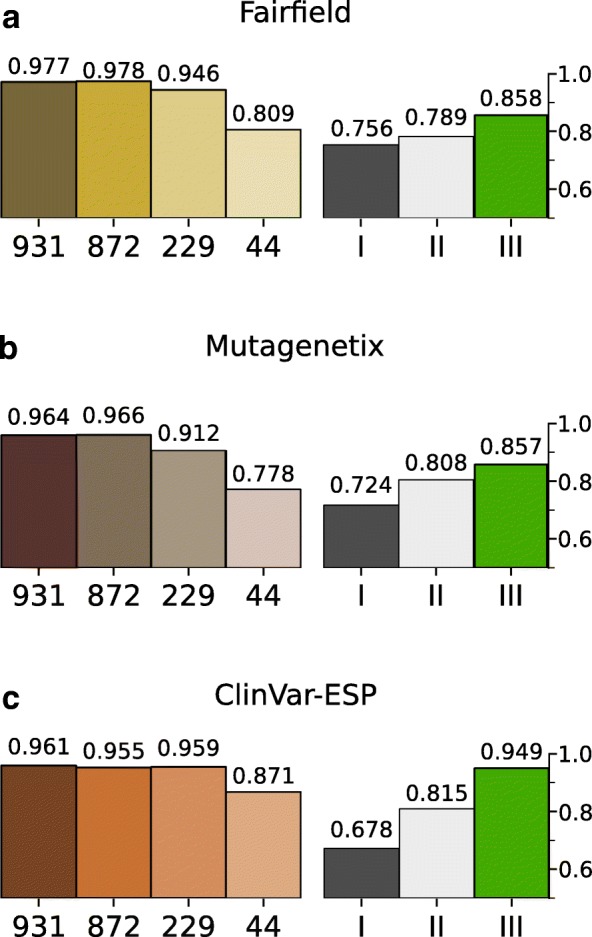
ROC-AUC scores of mCADD models evaluated on three different validation sets: the **a**) Fairfield, **b**) Mutagenetix and **c**) ClinVar-ESP data sets. The numbers below the bars indicate the number of annotations used during model training. Roman numbers indicate: I) the glire-PhastCons score, II) the vertebrate PhastCons score, and III) the hCADD* score. The numbers above the bars show the exact ROC-AUC of that particular model and validation set combination

To show that mCADD is capable of accurately scoring real data and not only differentiates between simulated and derived variants, we evaluated the different mCADD models on three independent validation sets (see Fig. 3). mCADD(931) and mCADD(872) perform extremely well on all three validation sets (ROC-AUC > 0.95) and hardly differ (see Fig. 3). mCADD(229) performs comparably well on the ClinVar-ESP data set and shows a drop in performance on the Fairfield and Mutagenetix data sets. The drop increases when fewer annotations are considered for training (mCADD(44)). All mCADD models and hCADD* perform better than the two conservation scores, except for mCADD(44) on the Mutagenetix data. On all validation sets, the hCADD* performance lays between the performances of mCADD(229) and mCADD(44) and has relatively good performance on the ClinVar-ESP data set.

### Species-specific CADD model improves performance

To learn whether it is necessary to develop a mouse-specific model, we additionally lifted all three validation data sets from mm10 to GRCh37 and annotated the variants with CADD v.1.3 C-scores. We took care to only lift variants which have the same reference allele, thus displaying the same nucleotide substitution. Some variants could not be lifted due to a missing homozygous region. Negative samples were more often not lifted than positive ones, i.e. the Fairfield data set loses 50 negative samples and 27 positive ones, the Mutagenetix data set loses 235 positive and 398 negative samples, and for the ClinVar-ESP data set we had to omit 5 positive sample and 103 negative ones, due to the requirement of having the same reference allele.

**Fig. 4:**
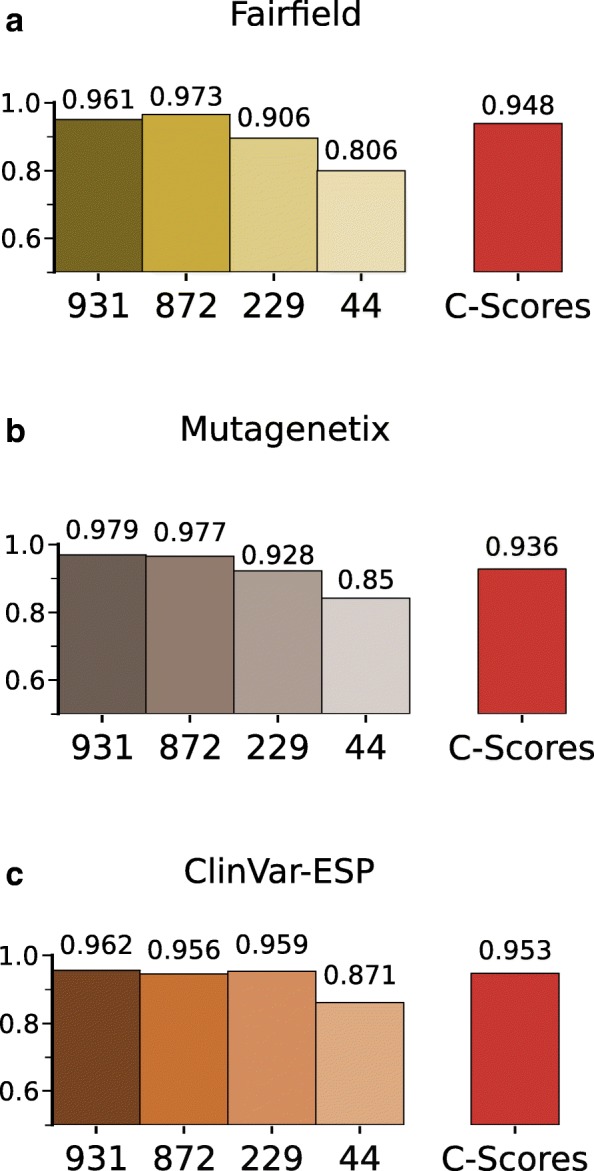
ROC-AUC scores of mCADD models and C-scores evaluated on three different validation sets (**a**) Fairfield, **b**) Mutagenetix, **c**) ClinVar-ESP) lifted from mouse to human. Arabic numbers underneath the bars indicate the number of annotations used for model training. The numbers above the bars show the exact ROC-AUC of that particular model and validation set combination

For the Fairfield data set, the performance of all mCADD models dropped due to the removal of 77 samples (see Fig. 4a). The C-scores perform between mCADD(229) and mCADD(872).

For the Mutagenetix data set, the mCADD models did not suffer from the removal of 633 SNVs, instead all computed ROC-AUCs increased (Fig. 4b). The C-scores perform again between mCADD(229) and mCADD(872).

For the Clinvar-ESP data set, the mCADD model performances are hardly affected (see Fig. 4c). Applied on the ClinVar-ESP data set, mCADD(229) performs better than C-scores.

Taken together, the species-specific mCADD model outperform lifting variants to human and using the hCADD model to score the variants, especially if considered that not every SNV can be easily lifted.

### Annotation weights are moderately correlated between mCADD and hCADD

**Fig. 5:**
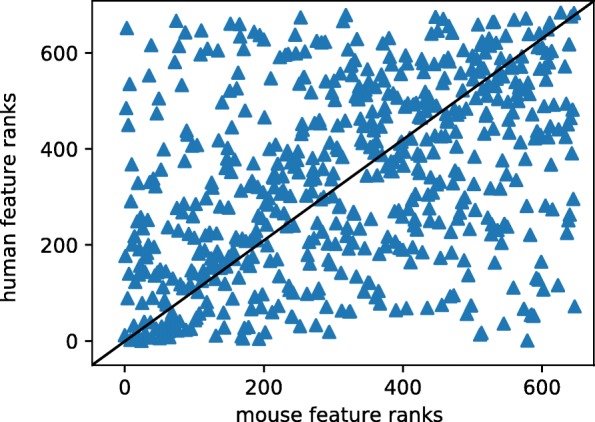
Comparing the ranks of the absolute weights assigned to annotations when training mCADD (horizontal axis) with those when training hCADD (vertical axis). A lower rank indicates an annotation with larger impact on the log-odds of a model

We examined whether different annotations are used by mCADD and hCADD. The absolutes of weights, assigned to each annotation by the logistic regressor, were ranked and the ranks of 595 annotations with a non-zero weight in both models were plotted against each other (see Fig. 5), having a Spearman’s rank correlation of 0.4.

Top-ranking mCADD annotations are enriched in combinations of DNA secondary structure predictions of DNAshape [23] (see Additional file 1: Table S4). Furthermore, predictions of intronic and intergenic regions seem to be important, together with the neutral evolution score of GERP++ (GERPN) [18].

Top-ranking hCADD annotations are PhastCons and PhyloP conservation scores, all based on different phylogenies. Of these, the most influential annotations are PhastCons scores based on a primate alignment [5, 19]. The second most important group of annotations are predictions on intronic regions.

The combination of primate-based PhastCons scores in hCADD with predicted VEP consequences indicating intronic and intergenic regions is similar to the combination of the same VEP consequences and the neutral evolution score of GERP++ in mCADD. From this, we conclude that the primate-based PhastCons scores are replaced by GERPN in mCADD.

Vertebrate-based PhastCons scores are ranked high for both mCADD and hCADD. Top ranked annotations in hCADD which are ranked low in mCADD are enriched in mammalian-based PhastCons and mammalian-based PhyloP scores. Vice versa, feature combinations with DNA secondary structure predictions are exclusively used by mCADD.

## Discussion

We demonstrated the possibility of creating a CADD-based model for the mouse genome, capable of predicting the deleteriousness of variants. We created a model trained on mouse data (mCADD) and evaluated it on a held-out test set and validation sets of phenotype altering SNVs. We compared the performance of our model to that of other metrics, such as conservation scores and the variant prioritization tool SIFT, as well as to C-scores for which we lifted the annotated variant locations to the human genome. We also compared performances on mouse test set variants to deleteriousness estimates of human test set variants, a.o. scored with a human CADD model that we trained ourselves (hCADD). As a final approach we trained a model on human data and evaluated it on mouse data (hCADD*).

Performances of mCADD and hCADD were very similar, with the mouse model performing better on the hold-out test sets. In addition, validation on three experimentally annotated data sets showed that the mCADD model is clearly capable of prioritizing deleteriousness of SNVs. Scoring lifted variants with hCADD performed reasonably well on these validation data sets, but less so than mCADD, whereas the generic hCADD* model had a consistent performance between mCADD(229) and mCADD(44). Together, this shows the importance of generating species-specific models when more annotations are available than only sequence specific ones, especially when lifting is not an option.

Evaluating the trained models on variants located in different genomic regions, we observed that mCADD and hCADD display the same trend, with increasing performance from non-coding to coding variants, and the best performance for missense mutations. Strikingly, mCADD, hCADD as well as other metrics all performed poorly on synonymous variants within coding regions.

We further assessed the annotation weightings in the human and mouse models. Despite a moderate correlation, both models rely on different annotations. This may explain the poorer performance of hCADD when evaluated on mouse data sets (i.e. hCADD*). Among the most important annotations are different conservation scores and/or combinations of these scores with VEP consequence annotations. It seems that hCADD relies relatively more on conservation scores than mCADD, while mCADD puts more emphasis on DNA structure predictions.

### Performance depends on genomic region

Previous studies indicated that performance of the CADD classifier is not constant over the entire genome [21, 22]. We also observed changing performances between the investigated genomic regions. This may be due to intrinsic differences in the SNVs, but it might also be due to a difference in the number of annotations between non-coding and coding regions. When evaluating the distribution of putative deleterious and benign SNVs across genomic regions (Additional file 1: Table S2), we find an imbalance in class labels of the held-out test set, but these do not explain the changes in performance. A striking difference in performance is found between the *translated missense* variants and *translated synonymous* variants. Annotations that help to differentiate between positive and negative missense mutations, such as SIFT, are not available for synonymous mutations. Hence, the main predictors for *translated synonymous* SNVs are the same as those for non-coding regions, namely different conservation scores, suggesting that the lack of meaningful annotations available for synonymous and other mutations limits the performance.

Note that CADD models are trained with putative benign and deleterious variants, as derived from the ancestor genome, and not with variants for which their effect is experimentally established. Although training variants are proxies, the trained CADD models perform extremely well on the experimentally validated SNVs as shown by the good performance on the validation sets. Apparently, the training variants are informative, and we, consequently, believe that the performances on the held-out test set can be interpreted at least qualitatively.

Together, this makes us believe that differences in observed performance between genomic regions are due to intrinsic properties of these regions such as the number of available annotations. This does, however, influence the applicability of any CADD-like model to prioritize disruptive SNVs truly genome wide.

### Models based on limited numbers of annotations can be predictive

One of the objectives of this study was to investigate the predictive power of CADD-like models in the case of incomplete annotation sets when compared to the human case. For that purpose, we defined four different sub annotation sets: all annotations (mCADD(931), hCADD(1000)), all but epigenetic and species-specific annotations (m/hCADD(872)), annotations including VEP’s (m/hCADD(229)), and annotations including only conservations scores (m/hCADD(44)).

The general trend is that mCADD models perform worse with fewer annotations, on the held-out test set as well as on the three validation sets. This is most pronounced for variants within coding regions. Differences in performance between mCADD(931) and mCADD(872) are negligible. For the Fairfield and Mutagenetix validation sets, mCADD(872) even performs better. The biggest drop in performance is observed between mCADD(872) and mCADD(229), even though the performance of mCADD(229) on all three validation sets is still above ROC-AUC > 0.91. These results indicate that a reliable model can be built, even if only very few annotations are known. Moreover, if only conservation scores and sequence features are available, it is still possible to outperform individual conservation scores.

hCADD shows a similar, but lower, trend, although the performance of hCADD(872) improves over that of hCADD(1000) using all subsets of the held-out test set. One of the main differences between mCADD and hCADD is that when generating training variants, mCADD uses an evolutionary older ancestor genome than hCADD. Thus, the time window over which mouse-derived variants have experienced purifying selection is longer than in the human case. Equally, substitution rates for the simulated SNVs are derived from evolutionary more distant ancestors, resulting in a larger proportion of deleterious SNVs in mouse than in human data. The impact of the evolutionary observed differences is, however, poorly understood and warrants further investigation.

### Limited interpretability of scores mapped between different species

An established method to evaluate different alleles in the genome of any species is to compare them with known orthologous regions in other species for which annotations are known. Although annotating lifted variants with human-based C-scores worked well, evaluating the same variants with a species-specific model gave better results. In addition, not every variant position in the validation sets could be annotated by C-scores as they have to be located in sequences that can be aligned to human. Further, similar variants in different species may differ in the phenotype they cause. This has to be considered for any comparative genomic analysis [24].

## Conclusions

We have shown that the CADD approach for prioritizing variants can be applied to non-human species, and that it is important to train species-specific models. Interestingly, not all original annotations used by CADD are necessary to achieve good performance: only conservation scores and VEP consequences of variants (the set of 229 annotations we explored) may suffice to make meaningful predictions. These annotations are available for many species. Nevertheless, if possible, adding additional annotations for coding regions will help to improve the trained models. Altogether, our work has shown that species-specific CADD models can be successfully trained, opening new possibilities for prioritizing variants in other less well-studied species.

## Methods

### Overview of the CADD approach

We construct a CADD model for mouse, mCADD, as well as a CADD model based on human data, here denoted by hCADD. In contrast to the original CADD approach, mCADD and hCADD are trained specifically on single nucleotide variants. We also construct a model trained on human data and evaluated it on mouse variants, which will be further referred to as hCADD*. The purpose of this model is to learn about the performance to be expected if one wants to evaluate variants for which no model exists and that cannot be lifted between genomes. The SNVs and their annotations used for hCADD and hCADD* originate from the data set used for CADD v.1.3. Annotations that are specific for insertions or deletions were removed from the data set. Briefly, the original CADD model [5] is trained to classify variants as belonging to the class of *simulated* or *derived* variants. To train the CADD model, simulated and derived variants were generated based on the human-chimpanzee ancestral genome and mutation rates derived from a 6-taxa primate alignment [25].

Derived variants are variant sites with respect to the ancestral genome that are fixed in the human lineage, or nearly fixed with a derived allele frequency of above 95% in the 1000 Genomes Project [5]. Due to the purifying selection they experienced, derived variants are assumed to be depleted in deleterious variants.

Next to observed derived variants, variants are simulated that do not occur in the human lineage. Hence, simulated variants did not experience purifying selection, therefore fitness reducing variants are not depleted in this group. All variants are annotated with a large number of genomic features, ranging from sequence features, conservation scores, variant effect predictor annotations to epigenetic measurements.

### Derived and simulated variants in mouse

Due to a lack of sufficient sequencing data of large, freely reproducing mouse populations, we focused on identifying differences between an inferred mouse-rat ancestral genome and the most recent mouse reference assembly (mm10) [26]. The mouse-rat ancestral genome is based on the EPO 17-eutherian-mammal alignments [25, 27, 28] (Additional file 1: Figure S2) provided by Ensembl release 83 [29]. In total we observed 33,622,843 sites with a derived allele in the mouse reference that were not adjacent to another variant site.

To generate an equal number of simulated variants we made use of the CADD variant simulator [5]. Based on the mm10 reference, it uses an empirical model of sequence evolution derived from the EPO 17-eutherian-mammal alignments, with CpG di-nucleotide specific rates and locally estimated mutation rates within windows of 100kb. Only SNVs with a known ancestral site were selected. In this way, we generated 33,615,003 SNVs. The final dataset contains an equal number of simulated variants, equally divided over 11 folds (10 for cross-validation and training, the remaining for testing), yielding a total of 67,229,998 SNVs. Additional file 1: Table S2 gives an overview of these SNVs and their distribution over different genomic regions.

### Genomic annotations

An overview of all annotations that we assembled for mouse can be found in Additional files 2, 3. Histone modifications, transcription factor binding sites, DNAase Seq peaks and RNAseq expression measurements were downloaded from ENCODE [16]. The mm10.60way vertebrate alignment was retrieved from the UCSC Genome Browser [30]. This multiple sequence alignment was used to calculate four different PhyloP and PhastCons scores based on differently sized sub alignments, in particular an 8-taxa Glire alignment, a 21-taxa Euarchontoglire alignment, a 40-taxa Placental alignment and a 60-taxa Vertebrate alignment (Additional file 1: Figure S1). PhyloP and PhastCons scores were computed without taking the mouse reference sequence into account. Furthermore, information about regulatory motifs, micro-RNA predictions (microRNA binding [31], microRNA targets [32]) and chromatin state predictions (ChromHMM [33]) were taken into account. GERP++ neutral evolution and rejected substitution scores, GERP Elements scores and GERP Elements *p*-values were taken from [18] and mapped from mm9 to mm10 via CrossMap [34]. All 5-mer combinations of the 4 nucleotides were generated and based on that the DNA secondary structure was predicted for each 5-mer [23]. Differences in the predicted scores for the reference 5-mer and alternative 5-mer at the investigated positions were used as annotation. Summaries of consequences predicted by the Ensembl Variant Effect Predictor (VEP v.87 [27]) were used in combination with other annotations to create additional composite annotations (Additional file 1: Table S1, and Supplementary Note, Additional file 2). Additional annotations that rely on a gene build such as the SIFT protein score, reference and alternative amino acid, variant position within a transcript and coding region are also generated by VEP v.87.

Human annotations were downloaded from the original CADD publication v.1.3. [5] (download: 17-2-2016). Annotations which are by definition only available for InDels were removed.

### Annotation subsets

From the annotations, four subsets were created of decreasing size and increasing likelihood of availability in non-human species (see Additional files 3 and 4 for a complete overview). The first set consists of all available annotations, i.e. 1000 for hCADD, 931 for mCADD and 902 for hCADD*. The annotations used to train hCADD* are those which can be meaningfully compared between mouse and human. The second subset has 872 annotations. It excludes all epigenetic annotations and species-specific ones, leaving annotations available for both mouse and human. The third subset incorporates 229 annotations, including conservation scores, nucleotide sequence features and VEP consequence/annotation combinations. Annotations specific for coding regions were excluded, with the exception of coding region-specific VEP consequence values. The fourth subset of 44 annotations can be entirely generated from the sequence information itself. It includes conservation scores and nucleotide sequence annotations, such as the GC% within a 75 bp window upstream and downstream of the variant position.

### Training and evaluating the mCADD model

The CADD model is centered on a logistic regressor trained to differentiate between simulated and derived variants. This was done using the logistic regression module of Graphlab v2.0.1 [35], the same tool the CADD authors have used since CADD v1.1. Before training we standardized the human and mouse data by dividing each feature by its standard deviation. We did not center the features, in order to preserve sparsity. The mouse data set was split into 11 partitions of equal size (6,111,818 SNVs). The 11th partition was used as held-out test set. On the remaining 10 partitions we performed 10-fold cross validation to determine the number of training iterations for the logistic regressor and the *L*_2_ regularization parameter. The cross validation results are shown in Additional file 1: Table S3. The final model was trained on the joined ten partitions with a maximum number of 100 iterations and a regularization parameter set to 0.1.

To obtain the human held-out test set, we selected 2,851,642 SNVs. Similar to the mouse case, this amounts to every 11th SNV from those available in the CADD v.1.3 data set. The hCADD and hCADD* models are trained with a maximum number of 10 iterations and an *L*_2_ regularization parameter of 1, to keep the settings as similar as possible to CADD v.1.3.

All model performances were evaluated with the area under the receiver operating characteristic (ROC-AUC). Trained classifiers were assessed based on their performances on their respective held-out test sets. These sets were further divided according to the genomic regions from which each variant originates. An overview and description of the resulting 7 subsets can be found in Additional file 1: Table S2.

We further evaluated the classifiers on three additional data sets: (*i*) 60 SNVs associated with changes in phenotype as obtained from an exome sequencing study of 91 mouse strains with Mendelian disorders (Fairfield data set) [36]; (*ii*) 481 *N*-ethyl-*N*-nitrosourea (ENU) induced SNVs (Mutagenetix data set) [37]; (*iii*) 9348 variant sites lifted from the ClinVar-ESP validation set utilized in CADD v.1.3 (ClinVar-ESP data set) [5]. Similar to the training data, all data sets were standardized but not centered, using the scaling factors for each annotation which were obtained from the whole mouse data set.

Data for the Fairfield validation set is provided by Additional file 1: Table S4 [38] of the Fairfield et al. publication. The Mutagenetix data set was provided by several labs and downloaded from the Mutagenetix data base [37, 39]. All data were checked for the reported reference allele and, in the case of uncertainty, manually verified with the records on the website. If the reported allele could not be found in close proximity of the reported genomic location, the variant was discarded. Both the Fairfield and Mutagenetix validation sets contain phenotype altering SNVs, therefore all of these were considered as potentially deleterious without differentiating between the exact nature of the phenotype change (positive data set). To find an equal number of variants that can be used as a negative data set, we made use of SNVs identified in 36 mouse strains from the Wellcome Trust Sanger’s Mouse Genomes Project [15], filtered for an allele frequency (AF) ≥ 90%. We sampled to have a matching number of negative SNVs for both data sets, we took care that the proportions of transcribed, synonymous and non-synonymous mutations are the same among the positive and negative SNVs.

The ClinVar-ESP data set contains curated variants from the ClinVar database [14] that were identified to have a pathogenic effect in human. As a negative set (5635 SNVs), variants from the Exome Sequencing Project (ESP) [40] were selected with a derived allele frequency of ≥ 5%. We lifted the variants from GRCh37 to mm10 and selected SNVs which introduce the same amino acid substitution or stop codon change in human and mouse.

### Analysis of model weights

The logistic regressor assigns weights (betas) to each annotation used for training. These weights indicate the effect of one unit change on the log odds of success of the trained model. A zero weight implies that the annotation is not used. We compared the weights assigned to each annotation by mCADD and hCADD to derive information about annotations of general importance for CADD-like models. As different regularization terms were applied in hCADD and mCADD, causing the beta’s to be on different scale, we compared ranks instead of weights. Ranks were computed for non-zero beta’s and based on the absolute weight. Annotations of mCADD and hCADD were compared with each other when they have a non-zero weight in both models. Three types of annotations were not identical between mouse and human, but considered comparable:

1. Primate-based PhastCons&PhyloP [19, 20] scores in hCADD were compared with glire-based PhastCons&PhyloP scores of mCADD. These are the smallest alignments used to compute conservation scores in both species.
2. Mammalia based PhastCons&PhyloP scores in hCADD were compared to scores based on a placentalia alignment for mCADD.
3. CHROMHMM [33] chromatin state predictions were mapped based on the overlap of their predicted consequences in human and mouse.

## Additional files

Additional file 1 Supplementary data. Supplementary data containing tables and figures with additional information about the used phylogenies and other data. (PDF 203 kb)

Additional file 2 Annotation Overview. Excel sheet containing all human and mouse annotations which were used to generate features for model training. (XLSX 16 kb)

Additional file 3 Annotation Overview. Excel sheet containing all mouse annotation sub sets which were used to generate features for model training. (XLSX 15 kb)

Additional file 4 Annotation Overview. Excel sheet containing all human annotation sub sets which were used to generate features for model training. (XLSX 15 kb)

## Abbreviations

AF: Allele frequency
CADD: Combined annotation dependent depletion
ENU: *N*-ethyl-*N*-nitrosourea (induces point mutations in the genome)
ESP: Exome sequencing project
GERPN: Neutral evolution score of GERP++
hCADD: CADD model for evaluation of SNVs that is trained on human data
hCADD*: CADD model for evaluation of mouse SNVs that is trained on human data
mCADD: CADD model for evaluation of SNVs that is trained on mouse data
ROC-AUC: Receiver operating characteristic
VEP: Ensembl variant effect predictor
