# Supplementary material for: Predicting variant deleteriousness in non-human species: applying the CADD approach in mouse
Source: BMC Bioinformatics. 2018 Oct 12;19:373. doi: 10.1186/s12859-018-2337-5 (PMC6186050; doi:10.1186/s12859-018-2337-5)
Supplement: Supplementary file 1 — Supplementary data. Supplementary data containing tables and figures with additional information about the used phylogenies and other data. (PDF 203 kb) [file 12859_2018_2337_MOESM1_ESM.pdf]

Predicting variant deleteriousness in non-human species: applying the CADD approach in mouse - Supplementary Data

Christian Groß*[1,2], Dick de Ridder†[2] and Marcel Reinders‡[1]

[1]Delft Bioinformatics Lab, University of Technology Delft
[2]Bioinformatics Group, Wageningen University & Research

July 27, 2018

1 Supplementary Note

Annotation pre-processing

To train mCADD and hCADD models only SNV were considered.
Differences between the inferred ancestor genome and the mouse reference were utilized as negative class for training. Differences were considered when they were not adjacent to another site that was different between the ancestor and reference. These mutations are directed back in time while simulated variants are orientated forward in time. Therefore annotations that are sensitive to these differences have to be swapped in the set of derived variants. Namely, the nucleotide reference and alternative columns (*Ref, Alt*), the amino acid substitutions (*nAA, oAA*) and the variant effect consequence predictions made by the ENSEMBL Variant Effect Predictor v87 for the labels (*STOP_Gained, STOP_LOST*).

Descriptions of the annotation labels can be found in (Supplementary Data 2). Missing data for the following annotations were zero imputed:

motifEHIPos, GerpRS, SIFTval, GerpRSpval, mirSVR-Score, mirSVR-E, mirSVR-Aln, targetScan, Expression, DNAseSig, H3K27ac, H3K4me1, H3K4me3,

*c.gross@tudelft.nl
†dick.deridder@wur.nl
‡M.J.T.Reinders@tudelft.nl

tOverlapMotifs, motifDist, motifECount, motifEScoreChng, TFBS, TFBSPeak, TFBSPeaksMax, cDNApos, relcDNApos, CDSpos, relCDSpos, protPos, relprotPos, Dst2Splice, Grantham

The following annotations were mean imputed based on the mean of the simulated variants:

GC, CpG, dnaRoll, dnaProT, dnaMGW, dnaHelT, GerpN, GerpS, GerpRS, euaPhCons, euaPhyloP, gPhCons, gPhyloP, minDistTSS, minDistTSE, plaPhCons, plaPhyloP, verPhCons, verPhyloP

For the following annotations, another category (*UD = undefined*) was introduced to indicate missing values:

Domain, Dst2SplType, SIFTcat, oAA, nAA

Missing values in the annotation (*isTv*) were replaced by 0.5.

For the set of following annotations, an indicator feature was created which is set to 0 if the annotation is defined and set to 1 if undefined:

mirSVR-Score, targetScan, cDNApos, CDSpos, protPos, SIFTval,Grantham, Dst2SplType_ACCEPTOR, Dst2SplType_DONOR

The annotations (*minDistTSE ,minDistTSS*) were capped at 10000.

The following annotations were log-transformed:

minDistTSE ,minDistTSS, GerpRS

All categorical annotations were OneHotEncoded. Further annotation combinations were created. Namely, all possible combinations of *Ref* and *Alt*, representing an annotation for each possible nucleotide substitution. The same was done for *nAA* and *oAA*, thus there is one annotation for each possible amino acid substitution. Lastly, combinations of the set of the following annotations were made with each of the 15 summarized consequences (Supplementary Data 2) of the Ensembl Variant Effect Predictor.

cDNApos, CDSpos, Dst2Splice, GerpS, GerpN, plaPhCons, plaPhyloP, minDistTSE, minDistTSS, euaPhCons, euaPhyloP, protPos, relcDNApos, relCDSpos, relprotPos, verPhCons, verPhyloP, dnaHelT, dnaMGW, dnaProT, dnaRoll, gPhCons, gPhyloP

2 Supplementary Tables

Table 1: VEP consequences are summarized in 15 categories. If multiple annotations exist for the same variant, the consequence is selected according to the displayed hierarchy, with STOP-GAINED being the most important and UNKNOWN the least important category.

**Hierarchy**	**Abbreviation**	**VEP Consequence categories**
1	SG	STOP-GAINED
2	CS	CANONICAL-SPLICE
3	NS	NON-SYNONYMOUS
4	SN	SYNONYMOUS
5	SL	STOP-LOST
6	S	SPLICE-SITE
7	U5	5PRIME-UTR
8	U3	3PRIME-UTR
9	R	REGULATORY
10	IG	INTERGENIC
11	NC	NONCODING-CHANGE
12	I	INTRONIC
13	UP	UPSTREAM
14	DN	DOWNSTREAM
15	O	UNKNOWN

Table 2: This table gives a description about the genomic regions which were selected to evaluate the mCADD and hCADD models. Underneath the Genomic region, the total number of SNVs located in that region is displayed. H=Human, M=Mouse

Genomic Region<br>Total number SNV	Description	Class distribution<br>Human	Class distribution<br>Mouse
entire genome<br>H:31,368,062, M:67,229,998	randomly selected SNVs taken from the entire genome.	Derived: 0.5<br>Simulated: 0.5	Derived: 0.5<br>Simulated: 0.5
not-transcribed<br>H:30,592,093, M:64,278,844	randomly selected SNVs which are located outside of known transcript regions.	Derived: 0.5<br>Simulated: 0.5	Derived: 0.5<br>Simulated: 0.5
transcribed<br>H:775,969, M:2,951,154	randomly selected SNVs which are located in known transcript regions.	Derived: 0.4<br>Simulated: 0.6	Derived: 0.46<br>Simulated: 0.54
transcribed-not translated<br>H:461,057, M:1,684,821	randomly selected SNVs which are located in transcript regions but not translated. (5'UTR, 3'UTR, Intron)	Derived: 0.47<br>Simulated: 0.53	Derived: 0.5<br>Simulated: 0.5
translated<br>H:314,912, M:1,266,333	randomly selected SNVs which are located in known translated regions (Exon).	Derived: 0.29<br>Simulated: 0.71	Derived: 0.42<br>Simulated: 0.58
translated-synonymous<br>H:126,103, M:625,183	randomly selected SNVs which are located in translated regions but do not code for a missense annotations with an associated SIFT value.	Derived: 0.41<br>Simulated: 0.59	Derived: 0.62<br>Simulated: 0.38
translated-missense<br>H:188,809, M:641,150	randomly selected SNVs in translated regions that have a missense annotation with an associated SIFT value.	Derived: 0.21<br>Simulated: 0.79	Derived: 0.23<br>Simulated: 0.77

Table 3: 10-fold cross validation performance of mCADD models. Each row is showing a different number of iterations, each column a different L_2-penalization.

**Iteration | L_2-Penalization**	**0.1**	**1**	**10**
10	Mean: 0.623<br>Std: 0.01	Mean: 0.625<br>Std: 0.011	Mean: 0.626<br>Std: 0.009
100	Mean: 0.668<br>Std: 0.001	Mean: 0.634<br>Std: 0.104	Mean:0.667<br>Std: 0.003
1000	Mean: 0.638<br>Std: 0.06	Mean: 0.638<br>Std: 0.076	Mean: 0.653<br>Std: 0.042

Table 4: Top performing predictors in hCADD and mCADD

top 10 mCADD	top 10 hCADD	top 10 hCADD and mCADD	hCADD>500 and 100>mCADD	hCADD<100 and 500<mCADD
GerpN	priPhCons	verPhCons	UPxdnaMGW	IGxmamPhyloP
IGxGerpN	mamPhCons		UPxdnaRoll	IGxmamPhCons
dnaRoll	verPhCons		DNxdnaMGW	cHmmTssA
SIFTval	verPhyloP		RxdnaHelT	RxmamPhCons
IxGerpN	mamPhyloP		IxdnaRoll	oAAxUD
IGxdnaRoll	priPhyloP			IND_protpos
dnaMGW	IxpriPhCons			mamPhCons
IxdnaRoll	IGxpriPhCons			nAAxUD
verPhCons	GerpS			IND_CDSpos
GC	IxverPhyloP			

3 Supplementary Figures

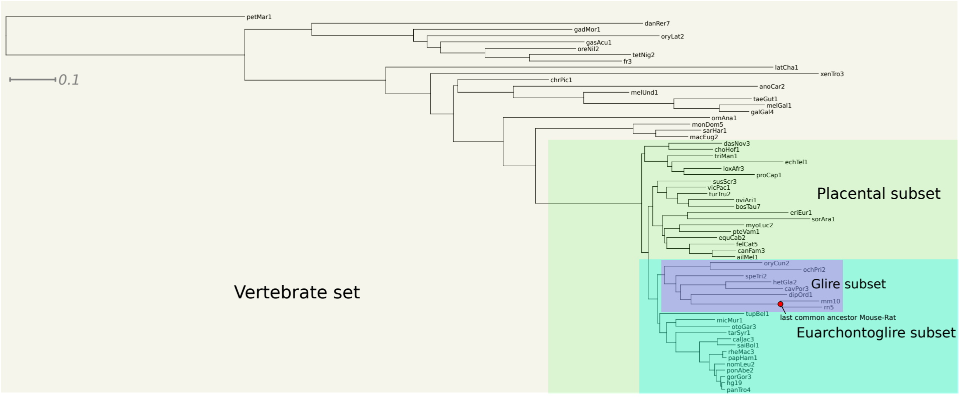

Figure 1: Phylogenetic tree, displaying the Vertebrate, Placental, Euarchontoglire and Glire sets which were used to compute PhastCon and PhyloP conservation scores. Furthermore, the last common ancestor between Mouse and Rat is indicated.

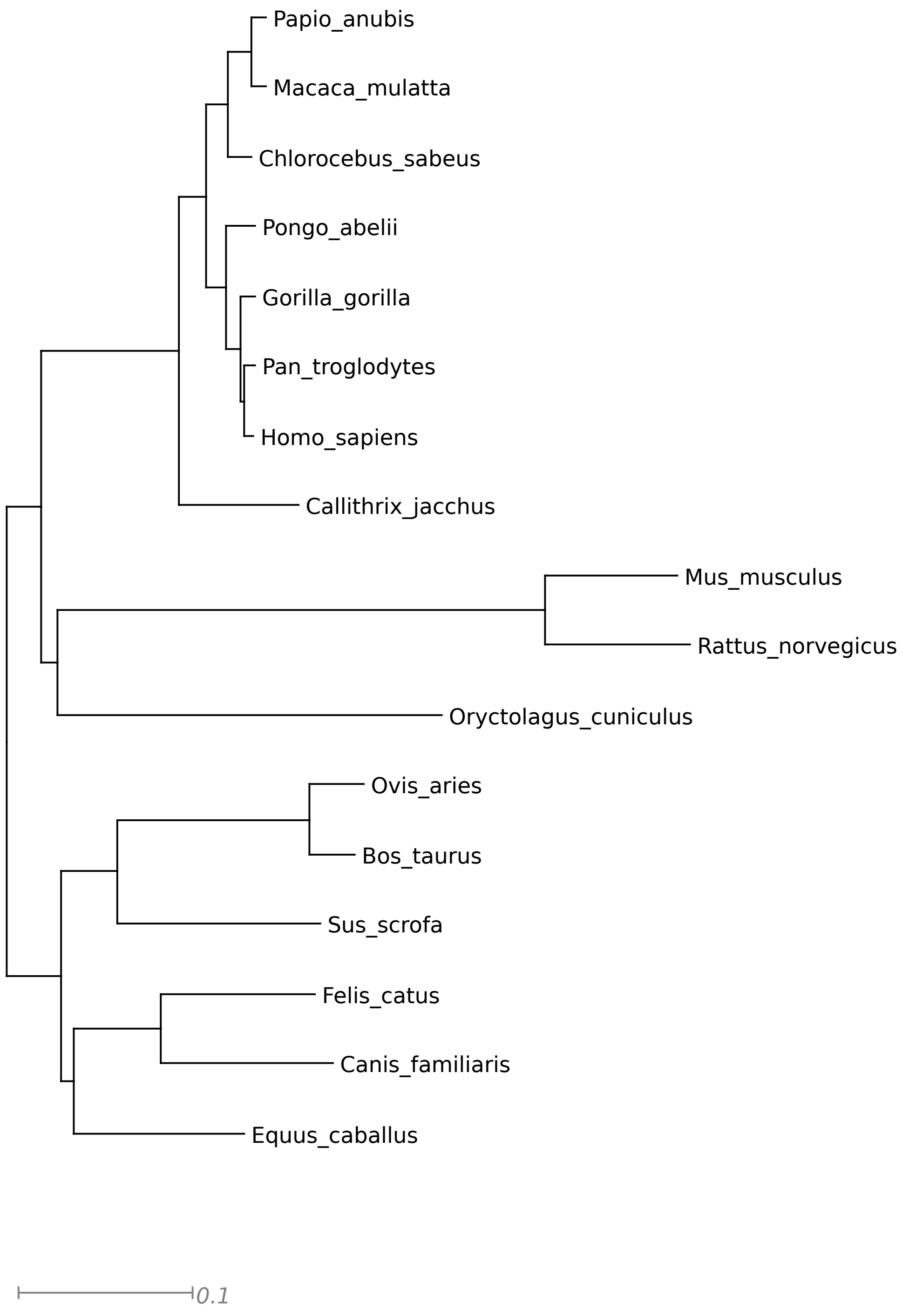

Figure 2: Phylogenetic tree, displaying the taxa used in the 17-eutherian-mammal EPO alignment. That alignment was used to infer the mouse ancestral sequence and to derive substitution rates to simulate variants.
